# Food security and diet quality in a racially diverse cohort of postpartum women in the USA

**DOI:** 10.1017/S0007114522001143

**Published:** 2023-02-14

**Authors:** Katelin M. Hudak, Sarah Gonzalez-Nahm, Tiange Liu, Sara E. Benjamin-Neelon

**Affiliations:** 1 Department of Health, Behavior and Society, Johns Hopkins Bloomberg School of Public Health, Baltimore, MD 21205, USA; 2 Lerner Center for Public Health Promotion, Johns Hopkins Bloomberg School of Public Health, Baltimore, MD, USA

**Keywords:** Food insecurity, Alcohol intake, Trans fat, Nurture, Alternate Healthy Eating Index

## Abstract

Food insecurity has been associated with poor diet, but few studies focused on the postpartum period – an important time for women’s health. We examined associations between food security and diet quality in postpartum women and assessed whether participation in federal food assistance programmes modified this potential relation. Using longitudinal data, we analysed the association between food security at 3 months postpartum and a modified Alternate Healthy Eating Index-2010 (AHEI) at 6 months postpartum (excluding alcohol). We conducted multivariable linear regressions examining associations between food security and AHEI. We assessed two food assistance programmes as potential effect modifiers. The sample included 363 postpartum women from the Nurture study, located in the Southeastern USA (2013–2017). Among women, 64·4 % were Black and 45·7 % had a high school diploma or less. We found no evidence of an interaction between food security and two federal food assistance programmes. In adjusted models, marginal, low and very low food security were not associated with AHEI. However, low (*β*: −0·64; 95 % CI −1·15, −0·13; *P* = 0·01) and very low (*β*: −0·57; 95 % CI −1·02, −0·13; *P* = 0·01) food security were associated with greater trans fat intake. Food security status was not associated with overall diet quality but was associated with higher trans fat (low and very low) and more moderate alcohol (marginal) intake. Future studies should assess the consistency and generalisability of these findings.

Approximately 11·5 % of Americans experienced food insecurity in 2018^(1)^. Having low household income, living in a female-headed household, having a higher number of children and being a racial minority have been positively associated with food insecurity^(2–4)^. Food insecurity – lacking consistent access to enough food for an active, healthy life^(1)^ – has been associated with an array of negative health outcomes in adults, including obesity^(5–7)^, poor cardiometabolic health^(8,9)^ and lower cognitive functioning^(10,11)^. Within a given household, family members may experience food insecurity differentially. For example, children may be partially shielded from food insecurity, while adults may be more severely affected^(1)^.

Diet quality is one possible mechanism through which food insecurity affects health outcomes. A recent study conducted by the US Department of Agriculture found that families in food insecure households purchased lower quality foods than food secure families^(12)^. Food insecurity has been associated with lower diet quality^(13–15)^, although the evidence has been mixed^(16–18)^. The association between food insecurity and obesity has been most consistently found among women^(15,19)^. Furthermore, there is evidence that this relation is especially salient in women with children^(20)^. In addition, maternal diet quality is a primary determinant of child diet quality^(21)^. Women directly influence children’s eating patterns through their own behaviours (i.e. modelling) and attitudes around food, as well as feeding styles^(22)^. Mothers’ influences on their children’s food preferences and eating patterns begin early in life – during pregnancy and infancy^(21,23)^. Therefore, understanding the connection between food security and maternal diet quality has implications not only for the health of women but also for children.

A few studies have examined associations between food security and diet quality among pregnant women. Laraia *et al*.^(24)^ found that food insecurity during pregnancy was positively associated with stress, disordered eating and dietary fat intake in women at 3 and 12 months postpartum. Nunnery *et al.* ^(25)^ found that higher levels of food insecurity were associated with lower availability and intake of fresh fruits and vegetables among low-income pregnant women from a southeast region of the USA. Conversely, using nationally representative data from the USA, Gamba *et al*.^(26)^ did not find a significant association between food security and diet quality among pregnant women.

The postpartum period is an important time in a woman’s life, during which the body undergoes unique changes in metabolism, haemodynamics and emotional status^(27)^. Healthy dietary patterns have been associated with a lower risk of postpartum depression and anxiety^(28)^, a more favourable lipid profile^(29)^ and a lower BMI in the postpartum period^(29)^. However, there is limited research examining food security and diet quality that is focused on the postpartum period. Glanville and Mcintyre^(30)^ assessed diet quality among single women living in the Atlantic region of Canada who had at least two children under the age of 14 years and found that although maternal diet quality was poor, it was not associated with food security. In contrast, Holben and Smith^(31)^ assessed differences in produce intake by food security status in a sample of women from Prince Edward Island, Canada, who had children 6 years and younger. They found that food insecure women consumed fewer fruits and vegetables than food secure women. Similarly, Yang *et al*.^(32)^ found that, at 18 months postpartum, food secure women in the city of Bradford in the United Kingdom consumed significantly more vegetables than food insecure women. However, this is the only study that has examined the relation between food security and diet quality in postpartum women in developed countries, despite the fact that it is an important and unique time for women^(33)^.

Federal food assistance programmes may play a role in shaping associations between food security and diet-related health in postpartum women. The Special Supplemental Nutrition Program for Women, Infants and Children (WIC) and the Supplemental Nutrition Assistance Program (SNAP) are two of the largest programmes in the USA. Both programmes provide food assistance to low-income families to help provide access to a higher-quality diet^(34,35)^. As such, WIC and SNAP have the potential to moderate a potential relation between food security and diet quality. Both WIC^(36,37)^ and SNAP^(38,39)^ participation are associated with higher food security. In addition, there is strong evidence that participation in WIC is positively associated with diet quality^(40–42)^. However, the connection that SNAP has with diet is less clear^(43)^. Evidence is mixed, with most studies suggesting that SNAP participants and income-eligible and higher-income non-participants are similar with respect to daily energetic and macronutrient intake, but that SNAP participants consume more fruit but less whole grains^(43–46)^.

We sought to examine longitudinal associations between food security and diet quality in a racially diverse sample of postpartum women. The secondary objective was to assess whether participation in either WIC or SNAP modified this potential relation. We hypothesised that lower food security would be associated with lower diet quality, and that participation in WIC or SNAP would attenuate this association.

## Methods

### Study design and population

We used data from the longitudinal Nurture study (2013–2017), an observational birth cohort of women and their infants from the southeastern USA. Recruitment has been described in detail elsewhere^(47)^. In brief, researchers recruited and enrolled women in mid- to late-pregnancy from a private prenatal clinic and a local health department. Researchers obtained written informed consent from each woman at recruitment during pregnancy. Women confirmed their willingness to participate shortly after delivery (online Supplementary Fig. S1). Women were eligible to participate if they were 20–36 weeks gestation with a singleton pregnancy, were at least 18 years of age, spoke and read English, planned to keep the baby and to stay in the area for the next 12 months. Women whose infants were born prior to 28 weeks’ gestation, had congenital anomalies that could affect growth and development, who had been in the hospital for 3 or more weeks or were not able to take food by mouth at the time of hospital discharge were excluded. Of the 860 women who enrolled during pregnancy, 666 women consented to participate for themselves and for their infants. Researchers conducted home visits at 3, 6, 9 and 12 months postpartum. Of the 381 women who completed FFQ during the 6-month home visit, 363 had complete data on food security at 3 months. The Nurture study was approved by the Duke University Medical Center Institutional Review Board (Pro 00036242).

### Exposure: food security at 3 months postpartum

Postpartum women completed the validated ten-item US Department of Agriculture Adult Food Security Survey Module^(48,49)^ at the 3-month study visit. The ten items ask about the food security of the postpartum women and other adults in the household. We modified the survey to ask about food security in the past 30 d rather than the past year because the postpartum period is dynamic, and we wanted to differentiate food security in the postpartum period from food security during pregnancy. We categorised postpartum women as having either high, marginal, low or very low food security using standard methods^(48)^. We classified women with zero affirmative responses as having high food security; 1–2 affirmative responses as marginal food security; 3–5 affirmative responses as low food security; and 6–10 affirmative responses as very low food security.

### Outcome: diet quality at 6 months postpartum

Women completed the Block FFQ^(50)^ at 6 months postpartum. The FFQ asked about usual food consumption in the past 3 months. The Alternate Healthy Eating Index-2010 (AHEI-2010) is based on US dietary guidelines and scores an individual’s diet to create a summary measure of overall diet quality^(51)^. Researchers at the Harvard School of Public Health developed the AHEI-2010 as an expansion of the Healthy Eating Index to assess not just diet quality but also chronic disease risk^(51,52)^. The food groups included in the score were chosen based on the 2010 dietary guidelines and their relation to lower chronic disease risk^(51,52)^. The AHEI-2010 applies to the general adult population^(51,52)^ and is not specific to the postpartum period. Even so, we opted to use the AHEI-2010 rather than other dietary indices because the AHEI-2010 is predictive of chronic disease risk^(51)^ and mortality^(53)^. Furthermore, previous studies with a focus on pregnant or postpartum women used a modified version of the AHEI that excludes alcohol^(54–56)^, and we followed this approach. We calculated the AHEI-2010 total score and component scores^(51)^ for all women. The modified AHEI-2010 total score has a maximum of 100, where higher scores indicate higher diet quality. The AHEI-2010 includes ten components, each of which has a maximum score of 10: (1) vegetables, (2) fruit, (3) whole grains, (4) nuts and legumes, (5) long-chain fatty acids (DHA and EPA), (6) PUFA, (7) red and processed meats, (8) trans fat, (9) Na and (10) sugar-sweetened beverages. Similarly, higher numbers in the component scores indicate a healthier level for the specific food or nutrient.

### Other variables

At study enrolment and at each home visit, women supplied information on socio-demographic characteristics and other variables. For the present analysis, we identified *a priori* maternal and household characteristics that have been associated with diet quality and food security: age, race (White, Black, other race/multiracial), education level (high school graduate or less, greater than high school), marital status (married, not married), number of children in the household, total weeks of breast-feeding and total daily kilocalories consumed^(13,26,55–58)^. Women also provided information on participation in WIC and SNAP at each home visit. WIC and SNAP have distinct goals and women can participate in both programmes simultaneously if they meet the eligibility criteria for both programmes individually. We used data on WIC and SNAP participation from the 3-month study visit for the current analysis.

### Statistical analysis

The analysis included 363 women who had complete food security and FFQ data. We used adjusted linear regression to examine the relation between food security and AHEI-2010 total and component scores in separate models. First, we assessed the connection between food security and AHEI-2010 with all covariates (age, race, education level, marital status, number of children in the household, total weeks of breast-feeding, total daily calories consumed) except indicators of participation in WIC or SNAP. Next, we added in controls for WIC and SNAP participation to examine whether programme participation affected results. To assess WIC and SNAP participation as potential modifiers, we conducted an overall *F*-test with three df. We also investigated the robustness of our results in two sensitivity analyses. First, we excluded eleven women who reported a total daily intake of 500 kilocalories or less and fourteen women who reported a total daily intake of 5000 calories or greater to see if results differed. Second, we repeated our main analysis while including the alcohol component in the AHEI-2010. We also assessed whether breast-feeding status altered the relation between marginal food security and alcohol consumption by restricting the sample to breast-feeding women only, as alcohol consumption is not recommended for breast-feeding women. We conducted all analyses using Stata 16.1 using a significance level of *α* = 0·05 (two-sided)^(60)^.

## Results

Women were predominantly Black (64·4 %), with White women comprising 24·0 % and other race or multiple race women comprising 11·6 % (Table 1). Fewer than half (45·7 %) had a high school education or less, and 60·9 % were married or living with a partner. Mean age was 28·2 (sd 5·9) years, and women breastfed 13·3 (sd 10·2) weeks, on average. The majority of women (66·7 %) had high food security, although 12·1 % experienced marginal, 8·3 % experienced low and 13·0 % experienced very low food security at 3 months postpartum. Almost all women (95·8 %) reported receiving WIC and 80·8 % reported receiving SNAP. At 6 months, women had a mean AHEI-2010 score of 41·5 (sd 11·2) (Table 2). Mean intake was 2040·1 (sd 1425·6) calories. When examining WIC and SNAP as potential modifiers, we found no evidence of an interaction between food security and WIC or SNAP. Therefore, we included both as covariates in adjusted models.

**Table 1.** Demographic characteristics of postpartum women in the Nurture study (Mean values and standard deviations; numbers and percentages, *n* 363)

Maternal characteristics	Mean	SD
Age in years	28·2	5·9
Children in household	2·3	1·3
Weeks any breast-feeding, birth to 6 months	13·3	10·2
	Percentage	*n*
Race		
White	24·0	87
Black	64·4	233
Other race/more than one race	11·6	42
Education		
≤ High school	45·7	166
> High school	54·3	197
Marital status		
Never married/divorced/separated	39·1	141
Married/living with partner	60·9	220
Food security status		
High	66·7	242
Marginal	12·1	44
Low	8·3	30
Very low	13·0	47
Received WIC, 0–3 months postpartum		
No	4·2	12
Yes	95·8	275
Received SNAP, 0–3 months postpartum		
No	19·2	55
Yes	80·8	232

WIC, Special Supplemental Nutrition Program for Women, Infants and Children; SNAP, Supplemental Nutrition Assistance Program.

**Table 2.** AHEI-2010 and AHEI-2010 component scores of postpartum women in Nurture study (Mean values and standard deviations, *n* 363)

Dietary outcomes	Mean	SD
AHEI-2010 total score	41·5	11·2
AHEI-2010 component scores		
Vegetable	3·2	2·7
Fruit	3·1	2·8
Whole grains	3·3	2·7
Nuts and legumes	5·0	4·0
Long-chain fatty acids (EPA and DHA)	3·1	2·7
PUFA	7·1	1·9
Trans fat	7·6	1·3
Red and processed meats	3·1	3·3
Na	4·6	3·4
Sugar-sweetened beverages and fruit juice	1·8	2·9

AHEI-2010, Alternate Healthy Eating Index-2010.

We compared models with and without controlling for participation in WIC and SNAP, and the results were similar. After adjustment for potential confounders and WIC and SNAP, marginal (*β*: 2·42; 95 % CI −1·32, 6·15; *P* = 0·20), low (*β* = −2·43; 95 % CI −6·60, 1·74; *P* = 0·25) and very low (*β* = −0·85; 95 % CI −4·48, 2·79; *P* = 0·65) food security were not associated with total AHEI-2010. However, women with low (*β* = −0·64; 95 % CI −1·15, −0·13; *P* = 0·01) and very low (*β* = −0·57; 95 % CI −1·02, −0·13; *P* = 0·01) food security had significantly lower AHEI-2010 trans fat component scores, compared to women with high food security (Table 3). In our sensitivity analysis, we did not observe any differences in results when we excluded women who reported low (500 kilocalories or less) or high values (5000 calories or greater) on the FFQ. Therefore, we present results with all women included. Results of our supplementary analysis in which we included the alcohol component in the AHEI-2010 were qualitatively similar to our main results (online Supplementary Table S1). Marginal food security was associated with moderate alcohol intake (*β*: 1·27; 95 % CI 0·33, 2·22; *P* = 0·01). When we restricted the sample to women who were not breast-feeding at 6 months postpartum, marginal food security was associated with a 1·77 (95 % CI0·69, 2·85; *P* = 0·002) higher AHEI-2010 alcohol score, compared to women with high food security. We did not find a significant association between marginal food security and the alcohol component score among women who were breast-feeding.

**Table 3.** Adjusted* linear regression of the association between food security† and AHEI-2010‡ and AHEI-2010 component scores§ (*β*-coefficients and 95 % confidence intervals)

	Without adjusting for WIC and SNAP			Adjusting for WIC and SNAP		
	*β*	95 % CI	*P*	*β*	95 % CI	*P*
AHEI-2010 total score						
Marginal	1·92	−1·61, 5·46	0·29	2·42	−1·32, 6·15	0·20
Low	−3·21	−7·20, 0·78	0·12	−2·43	−6·60, 1·74	0·25
Very low	−1·89	−5·18, 1·40	0·26	-0·85	−4·48, 2·79	0·65
AHEI-2010 component scores						
Vegetable						
Marginal	0·07	−0·82, 0·97	0·87	0·04	−0·87, 0·96	0·93
Low	0·40	−0·63, 1·42	0·45	0·50	−0·52, 1·53	0·33
Very low	−0·45	−1·29, 0·39	0·30	-0·44	−1·34, 0·46	0·34
Fruit						
Marginal	0·17	−0·81, 1·14	0·73	0·44	−0·55, 1·43	0·38
Low	−0·50	-1·62, 0·61	0·38	-0·34	−1·45, 0·76	0·54
Very low	0·03	−0·88, 0·95	0·95	0·06	−0·91, 1·03	0·90
Whole grains						
Marginal	0·30	−0·46, 1·05	0·44	0·40	−0·39, 1·18	0·32
Low	−0·03	−0·88, 0·82	0·94	0·16	−0·72, 1·03	0·73
Very low	0·14	−0·56, 0·84	0·70	0·16	−0·61, 0·92	0·69
Nuts and legumes						
Marginal	0·87	−0·35, 2·09	0·16	0·87	−0·47, 2·20	0·20
Low	−0·03	−1·41, 1·35	0·97	0·05	−1·45, 1·54	0·95
Very low	−0·76	−1·90, 0·38	0·19	−0·45	−1·75, 0·85	0·49
Long-chain fatty acids						
Marginal	0·47	−0·27, 1·21	0·21	0·42	−0·39, 1·22	0·31
Low	0·24	−0·60, 1·08	0·57	0·15	-0·75, 1·05	0·75
Very low	0·02	−0·67, 0·71	0·95	−0·06	−0·85, 0·72	0·87
PUFA						
Marginal	0·14	−0·55, 0·83	0·69	0·13	−0·60, 0·87	0·72
Low	0·00	−0·78, 0·78	0·10	0·12	−0·70, 0·94	0·77
Very low	−0·02	−0·66, 0·62	0·96	0·27	−0·44, 0·98	0·45
Trans fat						
Marginal	−0·14	−0·57, 0·30	0·54	0·02	−0·43, 0·48	0·92
Low	−0·62	−1·11, −0·13	0·01	−0·64	−1·15, −0·13	0·01
Very low	−0·63	−1·03, −0·23	0·002	−0·57	-1·02, −0·13	0·01
Red and processed meats						
Marginal	−0·40	−1·53, 0·72	0·48	−0·38	−1·53, 0·78	0·52
Low	−1·03	−2·30, 0·24	0·11	−0·68	−1·97, 0·60	0·30
Very low	−0·11	−1·15, 0·94	0·84	−0·09	−1·21, 1·04	0·88
Na						
Marginal	0·70	-0·62, 2·03	0·30	0·72	−0·70, 2·15	0·32
Low	−1·02	−2·48, 0·45	0·17	−1·26	−2·82, 0·30	0·11
Very low	−0·27	−1·50, 0·96	0·66	−0·10	−1·49, 1·30	0·89
Sugar-sweetened beverages and fruit juice						
Marginal	−0·10	−1·13, 0·92	0·84	−0·14	−1·20, 0·92	0·80
Low	−0·41	−1·57, 0·75	0·49	−0·32	−1·51, 0·86	0·59
Very low	0·28	−0·68, 1·25	0·56	0·58	−0·47, 1·63	0·27

AHEI-2010, Alternative Healthy Eating Index-2010; WIC, Special Supplemental Nutrition Program for Women, Infants and Children; SNAP, Supplemental Nutrition Assistance Program.

* Models adjusted for maternal age, race, education, marital status, number of children living in the household, weeks of any breast-feeding and mean daily calories. Models presented in the first two columns have a sample size of 330 postpartum women. Models presented in the last two columns also control for participation in WIC and SNAP and have a slighter lower sample size of 258 postpartum women.

† Food security refers to adult food security in the household, computed from the ten-item USDA Food Security Survey Module. Food security is a categorical variable, with high food security as the reference category.

‡ . Models presented here use a modified AHEI-2010 that excludes alcohol, with the total score having a maximum of 100. Includes juice in the sweetened beverage category.

§ Component scores range from 0 to 10.

## Discussion

In this cohort of racially diverse postpartum women from the southeastern USA, we found that food security status was not associated with overall diet quality in our sample of women. Food security was, however, associated with higher trans fat intake for women with low and very low food security. Moreover, we did not observe an interaction between WIC and food security or SNAP and food security. Therefore, the effect of food security did not vary by WIC or SNAP participation in our study. However, low variation in WIC and SNAP participation potentially hindered our capacity to identify significant moderation effects. This was contrary to our pre-specified hypothesis that women experiencing food insecurity would have lower diet quality, compared with women not experiencing food insecurity, and that WIC and SNAP participation would attenuate this association.

To our knowledge, no other studies have reviewed the connection between food security and diet quality in postpartum women in a high-income country setting. Even so, one study examined food insecurity and dietary diversity among pregnant women and lactating women in rural Malawi^(61)^. Kang *et al*.^(61)^ found that compared with food secure lactating women, food insecure women had significantly lower dietary diversity and were less likely to consume meat/fish or eggs. These results could potentially be relevant to the US context.

A higher AHEI-2010 score has been associated with a lower risk of gestational diabetes^(62,63)^, cancer^(64)^ and CVD^(64)^. A healthy diet in the postpartum period has been shown to have beneficial impacts on plasma inflammatory markers^(65)^, glucose regulation^(66)^ and reaching or maintaining a healthy body weight^(29,67,68)^ for women, and on the body fat percentage of infants^(69)^. Multiple studies have found a link between food insecurity and lower diet quality in women but not postpartum women specifically, except for one study that examined diet quality at 18 months postpartum in a sample of women from the city of Bradford in the United Kingdom^(13,25,32,70,71)^. We based our hypothesis on these prior studies. However, we focused on women in the early postpartum period whereas other studies assessed diet quality in pregnant women or women with older children. The postpartum period is a time of significant physiological and emotional changes^(72,73)^, making it a unique stage in a woman’s life. Our sample was also different in other aspects from prior studies. One study^(25)^ used a convenience sample from the southeastern USA, whereas two previous studies^(13,71)^ used large, nationally representative samples from the USA. Compared with national samples, a higher proportion of women in the Nurture study were Black and received less formal education^(74)^. In addition, other studies^(13,25,71)^ used household-level food security that included any children in the household. In contrast, because evidence suggests that adults are likely to experience food insecurity differently than children in the same household^(1,75–77)^, our food security measure focused on adults in the household. Furthermore, our analysis used longitudinal data, whereas most previous studies^(13,25,26,30,71)^ used cross-sectional data. Of note is the study by Laraia *et al*.^(24)^ that also used longitudinal data and focused on the postpartum period. Although study authors did not assess overall diet quality, they found that food insecurity during pregnancy was associated with a higher level of dietary fat intake at 3 and 12 months postpartum^(24)^.

We also found that, compared with women who reported high food security, women who experienced low or very low food security had significantly lower scores on the AHEI-2010 trans fat component, indicating that food insecure women consumed significantly more trans fat as a percentage of total energy intake. Similar to the present study, Mazidi and Vatanparasat^(78)^ found that serum trans fatty acids were higher in food insecure men and women from the US National Health and Nutrition Examination Survey (2009–2010). Dietary choices could explain the connection between food insecurity and higher trans fat consumption. Trans fats are produced when vegetable oils are partially hydrogenated in the food manufacturing process. Top sources of trans fats in the American diet include packaged snack foods, bakery products, margarines and deep-fried fast foods^(79)^. Substantial evidence indicates that consumption of trans fat increases the risk of CHD, sudden death from cardiac causes, all-cause mortality and metabolic changes that are linked with the metabolic syndrome^(79–83)^. Similarly, food insecurity has been associated with increased risk of the metabolic syndrome^(9)^ and clinical risk factors of cardiometabolic diseases, such as hypertension, hyperlipidaemia and poor glycaemic control^(84,85)^. Dietary choices – such as greater intake of trans fats – may be one of several potential mechanisms driving the connection between food insecurity and cardiometabolic diseases^(86,87)^.

Food insecurity may contribute to lower diet quality and specifically greater intake of trans fats through multiple pathways. Food insecurity is connected with increased stress and anxiety^(4,88,89)^. The physiological response to stress, namely increased cortisol and neuropeptide Y, may promote increased consumption of highly palatable items, such as packaged baked goods and salty snacks^(87,90)^. Using nationally representative data from the USA, multiple researchers have found that food insecurity was associated with a lower total Healthy Eating Index score, greater intake of added sugars, empty calories, sugar-sweetened beverages, high-fat dairy products and salty snacks^(13,91)^. Food insecurity has also been associated with greater consumption of fast food^(92,93)^. Packaged snack foods, bakery products and fast foods are top contributors of trans fats in the American diet^(79)^.

A second mechanism linking food insecurity and greater consumption of trans fat is related to the management of scarce household resources^(94,95)^. Food insecure households may decrease the variety and quality of the foods they purchase as a strategy to avoid hunger^(86)^. Highly processed, nutritionally poor foods tend to cost less than nutritionally rich foods like fruit, vegetables and fresh meats^(96)^. For example, Nunnery *et al*.^(25)^ found that very low food secure pregnant women had a significantly lower variety of fruits and vegetables available at home, compared with fully food secure women. Lower availability mediated lower intake of fruits and vegetables in women experiencing very low food security^(25)^. In the present analysis, we did not find evidence of a significant association between food insecurity and consumption of fruits, vegetables or whole grains. Thus, although theoretical and empirical reasoning suggests substituting highly processed, less healthy foods for nutritionally rich foods as a potential mechanism, we do not find support for this in the overall results of our study.

The mounting evidence on the health consequences of trans fats led the US Food and Drug Administration (FDA) in 2015 to determine that partially hydrogenated oils were no longer ‘Generally Recognized as Safe’. The FDA prohibited food manufacturers from adding partially hydrogenated oils to foods after 18 June 2018. The FDA provided time for foods produced before 18 June 2018 to work their way through the food system, and established 1 January 2020 as the final date of compliance^(97)^. Dietary data that we used in our analysis come from FFQ questionnaires that women completed prior to the FDA ban. Therefore, consumption of products containing trans fats likely will be higher in our sample than in samples collected in 2020 and onward. Even though trans fats should be removed from the American diet as of 2020^(97)^, it is of concern that postpartum women with low and very low food security consumed significantly more trans fats than women with high food security, as there is a strong link between trans fat and cardiometabolic disease^(79–81)^.

In addition, the results of our supplementary analysis indicate that marginally food secure postpartum women had a significantly higher score on the AHEI-2010 alcohol component, compared to women with high food security. A higher score on the AHEI-2010 component score indicates higher diet quality, and the AHEI-2010 ranks consumption of 0·5–1·5 alcoholic drinks/d with the highest score for women^(51)^. Thus, our findings indicate that marginal food security is linked with moderate alcohol consumption. There is limited research on the relation between food security and alcohol use. Food insecurity increased the odds of alcohol consumption among adults in Australia^(98)^ and among women with children aged 7–11 years in rural South Africa^(99)^. In contrast, in a sample of Wisconsin adults, food insecurity was associated with heavy alcohol use among men but not women^(100)^. These studies used a binary measure of food security and thus were unable to differentiate the connection between alcohol intake and marginal, low and very low food security. Our study adds to this limited body of research and indicates that marginal food security is associated with moderate levels of alcohol consumption. Future research should examine the relation between food security and alcohol consumption in other samples in the USA.

The American Academy of Pediatrics recommends that breast-feeding women minimise alcohol consumption, with specific guidelines to wait at least 2 h between alcohol consumption and breast-feeding^(101)^. To assess if breast-feeding status altered the relation between marginal food security and alcohol consumption, we repeated the analysis while stratifying the sample by breast-feeding status (online Supplementary Table S2). We found that food security status was not associated with the AHEI-2010 alcohol component score among breast-feeding women, but the association between marginal food security and the AHEI-2010 alcohol component was stronger when examining only women who were not breast-feeding. While it is encouraging that marginal food security was not significantly linked with alcohol consumption among breast-feeding women, the fact that this association is significant among non-breast-feeding women is of concern. Alcohol consumption by mothers of infants may create other potential risks for the infant, beyond the possibility of alcohol passing through breast milk. If moderate alcohol consumption is associated with decreased attentiveness and less optimal care for the infant, then even moderate alcohol consumption could pose risks to the infant. No identified studies have investigated this question, which warrants further study.

Our findings have implications for clinical care in the postpartum period. Food insecurity during the postpartum period can have serious consequences for the woman and her child. A multi-professional (e.g. obstetrician-gynaecologist, primary care practitioner, registered dietitian) follow-up with women in the postpartum period could provide more opportunities for women to discuss any health or dietary concerns. Furthermore, clinicians should be prepared to discuss food insecurity and could use the USDA six-item food security module^(102)^ to screen women for food insecurity.

There are several limitations to this study. First, with any study using self-reported intake, there is the potential for social desirability bias, in which people under- or over-report intake in general, or the consumption of specific foods in particular^(103)^. Misreporting consumption would bias the results towards the null hypothesis. That is, if women, especially those with less healthy diets, reported healthier consumption patterns that do not accurately reflect dietary intake, there would be less opportunity to identify a significant association between food security and diet quality. Second, Nurture women were not fully representative of the population in the southeastern USA. The racial composition of our sample included a higher representation of Black women. This limits generalisability of our findings. However, Black women are underrepresented in most US cohort studies^(104)^ so this study offers a unique perspective. Third, we assessed food security of adults within the household. Evidence suggests that women experience food insecurity to a greater extent than other members of the household^(105,106)^. Thus, the level of food insecurity of the Nurture women may be underestimated. However, we found 21·3 % of women in our sample were food insecure (low or very low food security) at 3 months postpartum, which is nearly twice the national average (11·5 %)^(1)^. Next, small sample sizes of women in the different categories of food insecurity (*n* < 50) potentially limited our power to detect significant associations. Similarly, nearly all women in our sample participated in WIC and most participated in SNAP, which may have hindered our ability to identify significant moderation effects. Future studies that include greater variability in programme participation could examine WIC and SNAP as potential effect modifiers.

In this longitudinal study of racially diverse postpartum women, we found that food insecurity was associated with higher consumption of trans fats, a particularly harmful fatty acid that is strongly connected with increased risk for cardiometabolic diseases. We also found that women experiencing marginal food security consumed more moderate amounts of alcohol. Food security, however, was not associated with overall diet quality in this sample of women. Also contrary to our hypothesis, we found no evidence that participation in WIC or SNAP modified the connection between food insecurity and overall diet quality or dietary components. Future studies should examine these research questions in other cohorts to assess the consistency and generalisability of our findings. Given high rates of food insecurity in the USA and the importance of the postpartum period, the role of food security on diet quality should be further examined. Future research can explore the potential mechanisms through which food security affects chronic disease risk and other diet-related health outcomes in women – considering the unique contribution of trans fat and alcohol.

## Supplementary material

For supplementary material accompanying this paper visit https://doi.org/10.1017/S0007114522001143. click here to view supplementary material
