# Supplementary material for: Food security and diet quality in a racially diverse cohort of postpartum women in the USA
Source: Br J Nutr. 2022 May 5;129(3):503–12. doi: 10.1017/S0007114522001143 (PMC9876811; doi:10.1017/S0007114522001143)
Supplement: Supplementary file 1 [file S0007114522001143sup.zip › S0007114522001143sup001.pdf]

Food Security and Diet Quality in a Racially Diverse Cohort of Postpartum Women in the United States

Katelin M. Hudak,[a] Sarah Gonzalez-Nahm,[a, b] Tiange Liu,[a, b] Sara E. Benjamin-Neelon[a, b]

[a] Department of Health, Behavior and Society, Johns Hopkins Bloomberg School of Public Health, Baltimore, Maryland, USA

[b] Lerner Center for Public Health Promotion, Johns Hopkins Bloomberg School of Public Health Baltimore, Maryland, USA

Corresponding author: Katelin M. Hudak, Hampton House 904, 624 N Broadway, Baltimore, MD 21205, USA, katelin.hudak@jhu.edu, 724.992.8303

Short title: Food Security and Diet in Postpartum Women

Key words: food insecurity; trans fat; Nurture; AHEI

Financial support: This work was supported by the National Institutes of Health (SEBN, grant number R01DK094841). The National Institutes of Health had no role in the design, analysis or writing of this article.

Conflict of interest: KMH, SGN, TL, SEBN – None.

Ethical Standards Disclosure: This study was conducted according to the guidelines laid down in the Declaration of Helsinki and all procedures involving research study participants were approved by the Duke University Medical Center Institutional Review Board (Pro 00036242). Written informed consent was obtained from all subjects.
