# Supplementary material for: Food security and diet quality in a racially diverse cohort of postpartum women in the USA
Source: Br J Nutr. 2022 May 5;129(3):503–12. doi: 10.1017/S0007114522001143 (PMC9876811; doi:10.1017/S0007114522001143)

**Supplementary Table 1. Adjusted^†^ linear regression of the association between food security^‡^ and AHEI-2010^§^ and AHEI-2010 component scores^|^: Including alcohol**

|  | **Without adjusting for WIC and SNAP** | | **Adjusting for WIC and SNAP** | |
| --- | --- | --- | --- | --- |
|  | **β (95% CI)** **^¶^** | ***P*** | **β (95% CI)** | ***P*** |
| AHEI-2010 total score |  |  |  |  |
| Marginal | 3.17 (-0.49, 6.82) | 0.09 | 3.74 (-0.11, 7.59) | 0.06 |
| Low | -3.65 (-7.78, 0.47) | 0.08 | -2.62 (-6.92, 1.67) | 0.23 |
| Very low | -1.94 (-5.34, 1.46) | 0.26 | -0.79 (-4.54, 2.95) | 0.68 |
| **AHEI-2010 component scores** | | | | |
| Vegetable |  |  |  |  |
| Marginal | 0.07 (-0.82, 0.97) | 0.87 | 0.04 (-0.87, 0.96) | 0.93 |
| Low | 0.40 (-0.63, 1.42) | 0.45 | 0.50 (-0.52, 1.53) | 0.33 |
| Very low | -0.45 (-1.29, 0.39) | 0.30 | -0.44 (-1.34, 0.46) | 0.34 |
| Fruit |  |  |  |  |
| Marginal | 0.17 (-0.81, 1.14) | 0.73 | 0.44 (-0.55, 1.43) | 0.38 |
| Low | -0.50 (-1.62, 0.61) | 0.38 | -0.34 (-1.45, 0.76) | 0.54 |
| Very low | 0.03 (-0.88, 0.95) | 0.95 | 0.06 (-0.91, 1.03) | 0.90 |
| Whole grains |  |  |  |  |
| Marginal | 0.30 (-0.46, 1.05) | 0.44 | 0.40 (-0.39, 1.18) | 0.32 |
| Low | -0.03 (-0.88, 0.82) | 0.94 | 0.16 (-0.72, 1.03) | 0.73 |
| Very low | 0.14 (-0.56, 0.84) | 0.70 | 0.16 (-0.61, 0.92) | 0.69 |
| Nuts and legumes |  |  |  |  |
| Marginal | 0.87 (-0.35, 2.09) | 0.16 | 0.87 (-0.47, 2.20) | 0.20 |
| Low | -0.03 (-1.41, 1.35) | 0.97 | 0.05 (-1.45, 1.54) | 0.95 |
| Very low | -0.76 (-1.90, 0.38) | 0.19 | -0.45 (-1.75, 0.85) | 0.49 |
| Long chain fatty acids |  |  |  |  |
| Marginal | 0.47 (-0.27, 1.21) | 0.21 | 0.42 (-0.39, 1.22) | 0.31 |
| Low | 0.24 (-0.60, 1.08) | 0.57 | 0.15 (-0.75, 1.05) | 0.75 |
| Very low | 0.02 (-0.67, 0.71) | 0.95 | -0.06 (-0.85, 0.72) | 0.87 |
| Polyunsaturated fatty acids | |  |  |  |
| Marginal | 0.14 (-0.55, 0.83) | 0.69 | 0.13 (-0.60, 0.87) | 0.72 |
| Low | 0.00 (-0.78, 0.78) | 0.10 | 0.12 (-0.70, 0.94) | 0.77 |
| Very low | -0.02 (-0.66, 0.62) | 0.96 | 0.27 (-0.44, 0.98) | 0.45 |
| Trans fat |  |  |  |  |
| Marginal | -0.14 (-0.57, 0.30) | 0.54 | 0.02 (-0.43, 0.48) | 0.92 |
| Low | **-0.62 (-1.11, -0.13)** | **0.01** | **-0.64 (-1.15, -0.13)** | **0.01** |
| Very low | **-0.63 (-1.03, -0.23)** | **0.002** | **-0.57 (-1.02, -0.13)** | **0.01** |
| Red and processed meats | |  |  |  |
| Marginal | -0.40 (-1.53, 0.72) | 0.48 | -0.38 (-1.53, 0.78) | 0.52 |
| Low | -1.03 (-2.30, 0.24) | 0.11 | -0.68 (-1.97, 0.60) | 0.30 |
| Very low | -0.11 (-1.15, 0.94) | 0.84 | -0.09 (-1.21, 1.04) | 0.88 |
| Sodium |  |  |  |  |
| Marginal | 0.70 (-0.62, 2.03) | 0.30 | 0.72 (-0.70, 2.15) | 0.32 |
| Low | -1.02 (-2.48, 0.45) | 0.17 | -1.26 (-2.82, 0.30) | 0.11 |
| Very low | -0.27 (-1.50, 0.96) | 0.66 | -0.10 (-1.49, 1.30) | 0.89 |
| Sugar sweetened beverages and fruit juice | |  |  |  |
| Marginal | -0.10 (-1.13, 0.92) | 0.84 | -0.14 (-1.20, 0.92) | 0.80 |
| Low | -0.41 (-1.57, 0.75) | 0.49 | -0.32 (-1.51, 0.86) | 0.59 |
| Very low | 0.28 (-0.68, 1.25) | 0.56 | 0.58 (-0.47, 1.63) | 0.27 |
| Alcohol^#^ |  |  |  |  |
| Marginal | 0.99 (-0.03, 2.01) | 0.06 | **1.27 (0.33, 2.22)** | **0.01** |
| Low | -0.97 (-2.28, 0.33) | 0.14 | -0.47 (-1.64, 0.69) | 0.42 |
| Very low | -0.10 (-1.18, 0.98) | 0.86 | 0.47 (-0.59, 1.54) | 0.38 |

^†^Models adjusted for maternal age, race, education, marital status, number of children living in the household, weeks of any breastfeeding, and mean daily calories. Models presented in the first two columns have a sample size of 330 postpartum women. Models presented in the last two columns also control for participation in WIC and SNAP and have a slighter lower sample size of 258 postpartum women.

**^‡^**Food security refers to adult food security in the household, computed from the 10-item USDA Food Security Survey Module. Food security is a categorical variable, with high food security as the reference category.

^§^AHEI-2010: Alternative Healthy Eating Index-2010. Models presented here use the full AHEI-2010 that includes alcohol, with the total score having a maximum of 110. Includes juice in the sweetened beverage category.

^|^Component scores range from 0 to 10.

^¶^CI: Confidence Interval

^#^Mean (standard deviation) alcohol consumption was 3.4 (2.6).

**Supplementary Table 2. Adjusted^†^ linear regression of the association between food security^‡^ and AHEI-2010^§^ and the AHEI-2010 alcohol component score^|^: Differences by breastfeeding status**

|  | **Without adjusting for WIC and SNAP** | | **Adjusting for WIC and SNAP** | |
| --- | --- | --- | --- | --- |
|  | **β (95% CI)** **^¶^** | ***P*** | **β (95% CI)** | ***P*** |
| **Breastfeeding women^††^** | |  |  |  |
| AHEI-2010 total score | |  |  |  |
| Marginal | 7.46 (-1.44, 16.36) | 0.10 | 7.92 (-1.50, 17.35) | 0.10 |
| Low | -2.88 (-14.14, 8.39) | 0.61 | -3.02 (-16.65, 10.61) | 0.66 |
| Very low | -2.02 (-8.91, 4.86) | 0.56 | -0.23 (-9.29, 8.84) | 0.96 |
| AHEI-2010 alcohol component score | | | | |
| Marginal | -0.36 (-3.17, 2.44) | 0.80 | -0.47 (-2.59, 1.66) | 0.65 |
| Low | -1.16 (-5.40, 3.09) | 0.59 | -0.065 (-3.41, 3.28) | 0.97 |
| Very low | -1.48 (-3.81, 0.84) | 0.21 | -0.58 (-3.26, 2.10) | 0.66 |
| **Non-breastfeeding women** | |  |  |  |
| AHEI-2010 total score | |  |  |  |
| Marginal | 3.64 (-0.61, 7.89) | 0.09 | 3.17 (-1.30, 7.64) | 0.16 |
| Low | -2.04 (-6.72, 2.63) | 0.39 | -1.95(-6.72, 2.82) | 0.42 |
| Very low | -1.11 (-5.25, 3.03) | 0.60 | -0.57 (-4.97, 3.83) | 0.80 |
| AHEI-2010 alcohol component score | | |  |  |
| Marginal | **1.99 (0.97, 3.01)** | **0.0001** | **1.770 (0.69, 2.85)** | **0.002** |
| Low | -0.48 (-1.71, 0.76) | 0.45 | -0.519 (-1.77, 0.73) | 0.41 |
| Very low | 0.67 ([-0.50, 1.84) | 0.26 | 0.744 (-0.48, 1.97) | 0.23 |

^†^Models adjusted for maternal age, race, education, marital status, number of children living in the household, weeks of any breastfeeding, and mean daily calories. Models presented in the first two columns have a sample size of 330 postpartum women. Models presented in the last two columns also control for participation in WIC and SNAP and have a slighter lower sample size of 258 postpartum women.

**^‡^**Food security refers to adult food security in the household, computed from the 10-item USDA Food Security Survey Module. Food security is a categorical variable, with high food security as the reference category.

^§^AHEI-2010: Alternative Healthy Eating Index-2010. Models presented here use the original AHEI-2010 that includes alcohol, with a total score has a maximum of 110. Includes juice in the sweetened beverage category.

^|^Component scores range from 0 to 10.

^¶^CI: Confidence Interval

^††^ The sample size for women who are breastfeeding is 103 when not adjusting for WIC and SNAP participation and drops to 49 when controlling for WIC and SNAP participation.

**^‡‡^** The sample size for women who are not breastfeeding is 226 when not adjusting for WIC and SNAP participation and 208 when controlling for WIC and SNAP participation.

**Supplementary Figure 1**


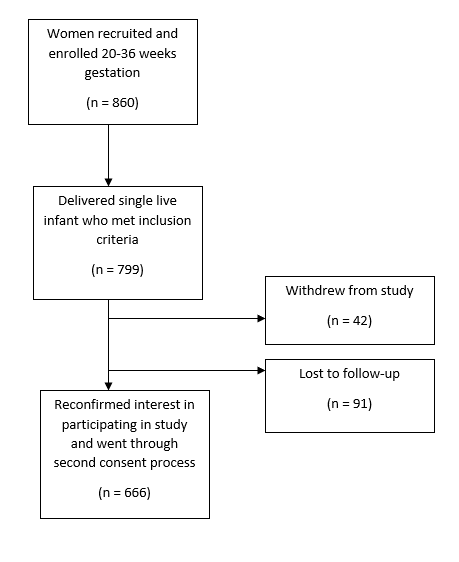

Supplement: Supplementary file 1 [file S0007114522001143sup.zip › S0007114522001143sup002.docx]
